# ﻿Revisiting the type species of the genus *Homidia* (Collembola, Entomobryidae)

**DOI:** 10.3897/zookeys.1176.109104

**Published:** 2023-08-22

**Authors:** Guo-Qiang Zhang, Yu-Xin Zhao, Feng Zhang

**Affiliations:** 1 Department of Entomology, College of Plant Protection, Nanjing Agricultural University, Nanjing 210095, China Nanjing Agricultural University Nanjing China

**Keywords:** DNA barcode, *Homidiacingula*, Southeast Asia, southwestern China, taxonomy

## Abstract

*Homidiacingula* Börner, 1906, the type species of the genus *Homidia* Börner, 1906, is widespread from India to Southeast Asia, but its detailed morphological characteristics have not yet been described. We examined the morphology of specimens of *H.cingula* from Indonesia and southwestern China and confirmed their conspecific status by comparing their DNA barcoding sequences. We also compared the morphology of *H.cingula* with other two closely related species, confirming the valid species status of *H.subcingula* Denis, 1948. Our study provides new taxonomic and molecular data for the genus *Homidia*.

## ﻿Introduction

The genus *Homidia* Börner, 1906 (Collembola, Entomobryidae, Entomobryinae) was first described as a subgenus of *Entomobrya* Rondani, 1861 (Börner 1906). It was later recognized as a distinct genus by Denis (1929). *Homidia*, with 76 reported species, is widely distributed in the Northern Hemisphere, particularly in East and Southeast Asia (Bellinger et al. 1996–2023). *Homidia* can be easily distinguished from other genera of Entomobryinae by several key characteristics, including 8+8 eyes, dental spines in adults, a subapical mucronal tooth larger than the apical one, and eyebrow-like macrochaetae on the anterior part of the fourth abdominal segment (Börner 1906; Szeptycki 1973).

The type species of the genus, *Homidiacingula* Börner, 1906, was originally described from Java (Buiterizor) and has since been recorded in India, Bangladesh, Thailand, Malaya, Singapore, Sumatra, and Vietnam (Yoshii 1989). *Homidiacingula* is characterized by its distinctive colour pattern, which includes pigmented abdominal segments II and III. This colour pattern is also seen in three other related species: *Entomobryakali* Imms, 1912 from India, *Homidiasubcingula* Denis, 1948 from Vietnam, and *Homidiaglassa* Nguyen, 2001 also from Vietnam. Handschin (1925) initially questioned whether *H.kali* was a synonym of *H.cingula*, and this was later confirmed by Mitra (1976) who re-examined the syntypes of *E.kali*. However, these species have notable differences in colour pattern based on their original descriptions (Table 1): thoracic patches and broad band on Abd. IV posteriorly in *H.cingula*; narrow band on Abd. IV posteriorly and transverse band on Abd. V in *H.subcingula*; and a pair of small metathoracic patches in *H.glassa*. Although Denis (1929) noted differences between *H.cingula* and *H.subcingula* (such as a broad vs narrow stripe on the posterior part of the fourth abdominal segment), Mitra (1976) suggested that *H.cingula* may represent juveniles and *H.subcingula* the darker form of adults. Nguyen (2001) only identified one difference between *H.subcingula* and *H.glassa*: two small patches on the metathorax in the latter. Unfortunately, the type material of *H.cingula* was destroyed (Weidner 1962). Further examination of the type species is essential to resolve the taxonomic uncertainties surrounding these species and to improve our understanding of the genus *Homidia*.

This study focuses on specimens of *H.cingula* collected from Indonesia (Java, Sulawesi) and China, as well as the types of *H.subcingula*. We also employ molecular barcoding techniques to obtain genetic sequences for *H.cingula* specimens from Java and China and compare their genetic distances. A detailed description of *H.cingula* is provided.

## ﻿Materials and methods

### ﻿Morphological examination

Juvenile and adult specimens were cleared in lactic acid, mounted in Marc André II solution, and studied using Leica DMLB and Nikon 80i microscopes. Illustrations were enhanced in Adobe Photoshop CS5. Dorsal body chaetae nomenclature follows Szeptycki (1979), Zhang and Deharveng (2015), and Zhang et al. (2019), labial palp nomenclature follows Fjellberg (1999), and labial chaetae nomenclature follows Gisin (1967). The dorsal chaetotaxy is given per half-tergite in the descriptions; the solid and hollow circles represent the primary and secondary chaetae, respectively.

### ﻿Abbreviations used in this study

**Th. I–III** thoracic segment I–III;

**Abd. I–VI** abdominal segment I–VI;

**Ant. I–IV** antennal segment I–IV;

**mac** macrochaeta(-ae);

**mes** mesochaetae(-ae);

**mic** microchaeta(-ae);

**ms** S-microchaeta(-ae) (microsensillum);

**sens** ordinary S-chaeta(-ae) on terga;

**NJAU** Nanjing Agricultural University;

**MNHN**Museum National d’Histoire Naturelle;

**NCBI** National Center for Biotechnology Information.

### ﻿DNA barcoding

DNA was extracted using an Ezup Column Animal Genomic DNA Purification Kit (Sangon Biotech, Shanghai, China) following the manufacturer’s standard protocols. Primers used were LCO1490/HCO2198, which are commonly used for metazoans (Folmer et al. 1994). PCR amplification of mitochondrial COI was performed in 25 μL volumes containing 12.5 μL of Premix Taq (TaKaRa Taq v. 2.0 plus dye), 1.25 μL of each primer, 8 μL of ddH_2_O, 2 μL of template DNA, with PCR programs following Zhang et al. (2014). All PCR products were checked on a 1% agarose gel. Successful products were purified and sequenced in both directions by Majorbio (Shanghai, China) on an ABI 3730XL DNA Analyser (Applied Biosystems). COI sequences for the remaining species were obtained from the NCBI (https://www.ncbi.nlm.nih.gov/). Sequences were preliminarily aligned using MAFFT v. 7.450 by the L-INS-I strategy (Katoh and Standley 2013) and corrected manually, with a final 658-bp alignment. Neighbour-joining (NJ) tree and Kimura 2-parameter (K2P; Kimura 1980) distances were calculated in MEGA v. 7.0 (Kumar et al. 2016). Node supports were evaluated through 1,000 bootstrap replications.

## ﻿Taxonomy

**Order Entomobryomorpha Börner, 1913**

**Family Entomobryidae Schäffer, 1896**

**Genus *Homidia* Börner, 1906**

***Homidiacingula* Börner, 1906**

**Entomobrya (Homidia) cingula Börner, 1906**

### ﻿ Entomobrya kali

Taxon classificationAnimaliaCollembolaEntomobryidae

Imms, 1912

254EA961-2744-5452-B446-6C87C50D0EFA

Figs 1–2 , 3–14 , 15–19 , 20–22 , Table 1

#### Type locality.

Buitenzorg, Bogor, Java Province, Indonesia.

#### Materials examined.

Indonesia • 2 adult females, subadult and 3 juveniles on slide, and four in alcohol; South Sulawesi Province, Kabupaten Bone, Watampone, near Lampo spring; 17 Jul. 1986; Anne Bedos leg.; in litter; sample # Indo-166; four specimens on slide deposited in NJAU and others in MNHN. Indonesia • 1 in alcohol; Jawa Timur; 2 Jul. 2001; Villemant & Daugeron leg.; sample # 03255D01_JAVA05CV03; deposited in MNHN. China • 2 females on slide and 3 in alcohol; Yunnan Province; 26.643°N, 98.905°E; 1,149 m a.s.l.; 11 Oct. 2014; C-Y Qin leg.; in litter; sample # 14YN2. China • 5 in alcohol; Yunnan Province; 27.007°N, 98.869°E; 1,199 m a.s.l.; 12 Oct. 2014; C-Y Qin leg.; sample # 14YN3. All Chinese material deposited in NJAU.

#### Redescription.

**Figures 1, 2.**
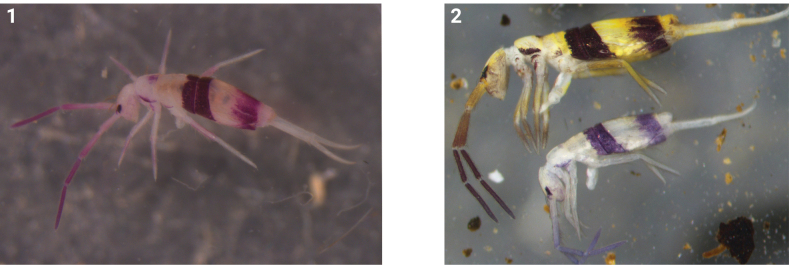
Habitus of *Homidiacingula* Börner, 1906 preserved in ethanol **1** from South Sulawesi (Indonesia) **2** from Yunnan (China).

Body length up to 2.38 mm. Ground colour pale yellow or pale. Antenna gradually darker towards tip. Eye patches dark blue. Th. II with lateral strips and a small patch on the postero-middle part. Th. III with very pale lateral strips. Coxae and femora weakly pigmented. Two transverse dark bands on Abd. II and III. Posterior half of Abd. IV pigmented (Figs 1, 2).

**Figures 3–14.**
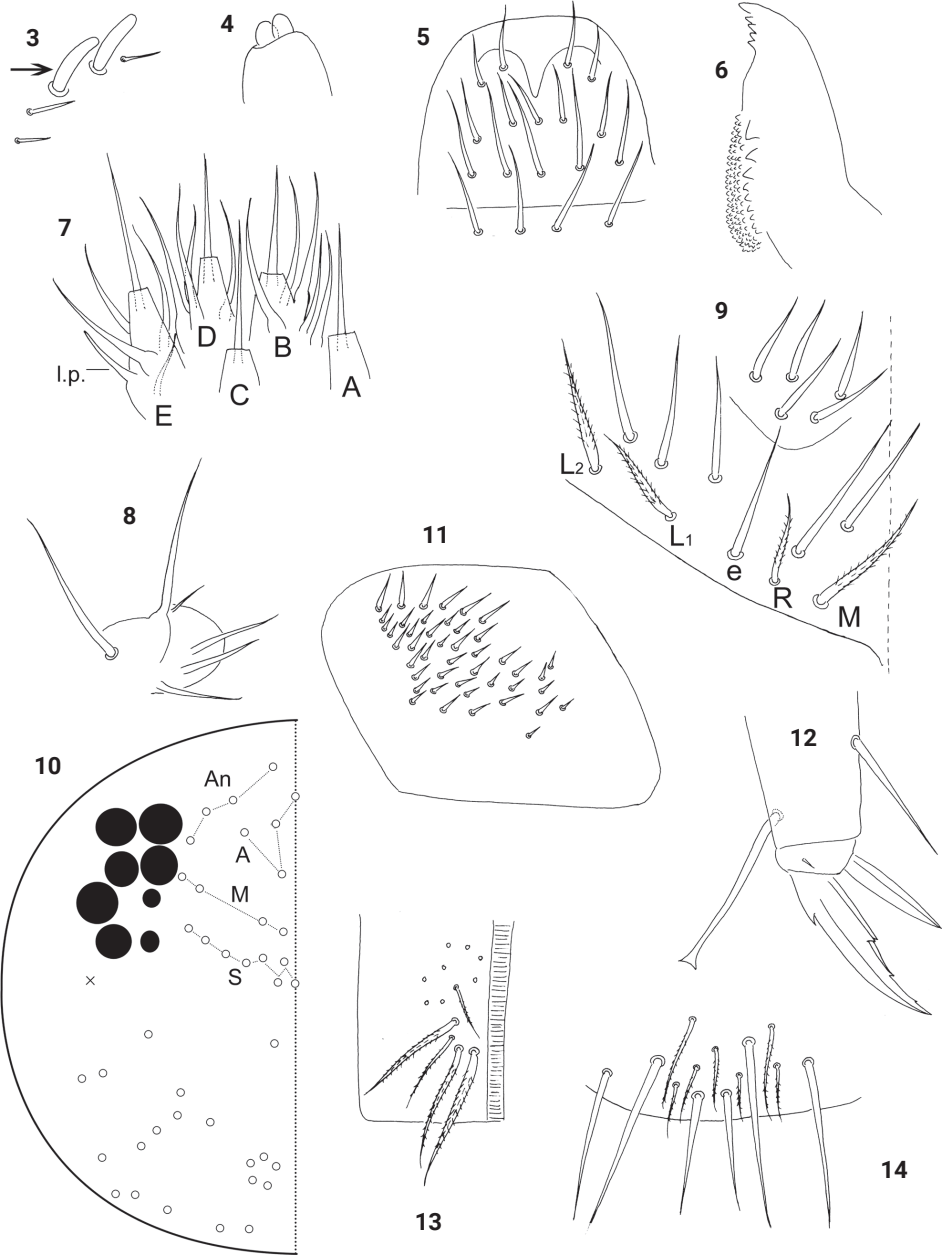
*Homidiacingula***3**Ant. III organ **4** Antennal apical bulb **5** labrum **6** right mandible **7** labial palp **8** maxillary outer lobe **9** labial chaetae **10** dorsal cephalic chaetotaxy **11** trochanteral organ **12** hind claw **13** ventral tube, anterior face **14** ventral tube, distal part of posterior face.

Antenna 2.5–3.0 times as long as cephalic diagonal. Antennal segments ratio as I: II: III: IV = 1: 1.2–1.3: 1.2–1.3: 1.6–1.9. Smooth straight mic at antennal base three dorsal and three ventral on Ant. I, one external, one internal and one ventral on Ant. II and absent on Ant. III and IV. Ant. III organ with two rod-like sensilla (Fig. 3). Ant IV with apical bulb bilobed (Fig. 4).

Eyes 8+8. Labral papillae absent. Labral intrusion deeply V-shaped. Prelabral and labral chaetae 4/ 5, 5, 4, all smooth; prelabral chaetae and chaetae of the first row longer than others (Fig. 5). Mandibles with 4+5 teeth (Fig. 6). Five labial papillae A–E with 0, 5, 0, 4, 4 guard chaetae, respectively; lateral process of labial palp thinner than normal chaetae, with tip not reaching apex of papilla E (Fig. 7). Subapical seta of maxillary outer lobe subequal to the apical one; four smooth sublobal hairs on maxillary outer lobe and the lateral one much smaller than others (Fig. 8). Labial base as MReL_1_L_2_; chaeta e smooth; proximal area with five smooth chaetae (Fig. 9); modified (leaf-like) chaetae absent on the ventral side. Cephalic dorsal chaetotaxy with four antennal (An), four anterior (A), four medio-ocular (M), and eight sutural (S) chaetae (Fig. 10).

**Figures 15–19.**
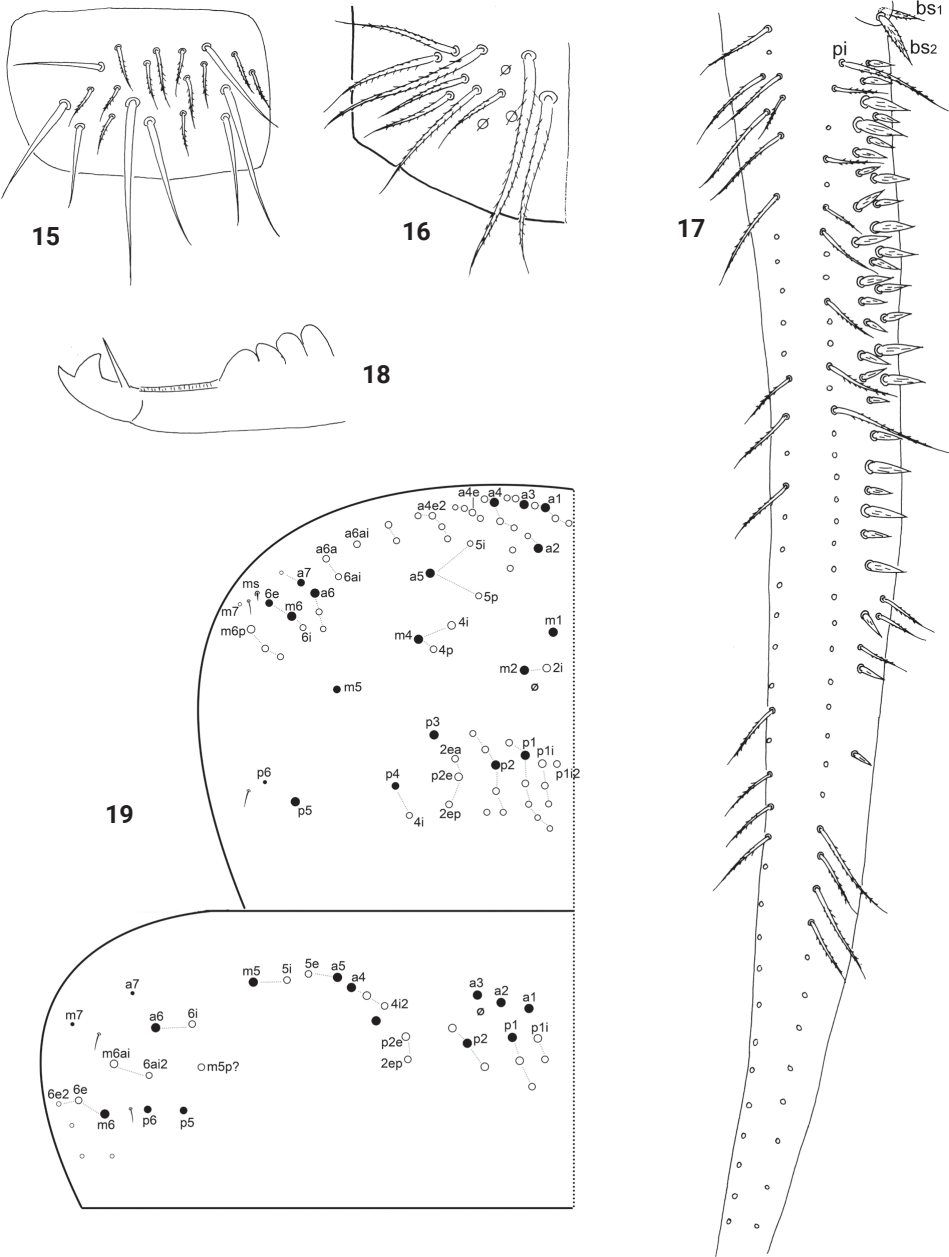
*Homidiacingula***15** lateral flap of ventral tube **16** manubrial plaque **17** posterior face of dens **18** mucro **19** thoracic chaetotaxy.

Coxal macrochaetal formula as 3/4+1, 3/4+2. Trochanteral organ with 31–35 smooth, spine-like chaetae (Fig. 11). Unguis with four inner and two lateral teeth; distal inner tooth extremely tiny. Unguiculus acuminate with outer edge smooth. Tenent hair clavate, subequal to unguis (Fig. 12). Abd. IV 4.4–5.1 times as long as Abd. III along dorsal midline. Tenaculum with 4+4 teeth and one large striate chaeta. Ventral tube anteriorly with 9–13 ciliate chaetae on each side, three of them mac (Fig. 13); posteriorly with numerous ciliate chaetae and six distal smooth ones (Fig. 14); each lateral flap with 8–10 smooth and 7–12 ciliate chaetae (Fig. 15). Manubrial plaque with three pseudopores and 7–10 ciliate chaetae (Fig. 16). Posterior face of dens with two longitudinal rows of chaetae; 27–35 spines present internal to the inner row of chaetae; two basal chaetae (following Szeptycki 1973) spiny and multilaterally ciliate, bs_1_ slightly shorter than bs_2_; proximal-inner seta (pi) ciliate, apparently thinner and much longer than bs (Fig. 17). Mucro bidentate with subapical tooth much larger than apical one (Fig. 18).

**Figures 20–22.**
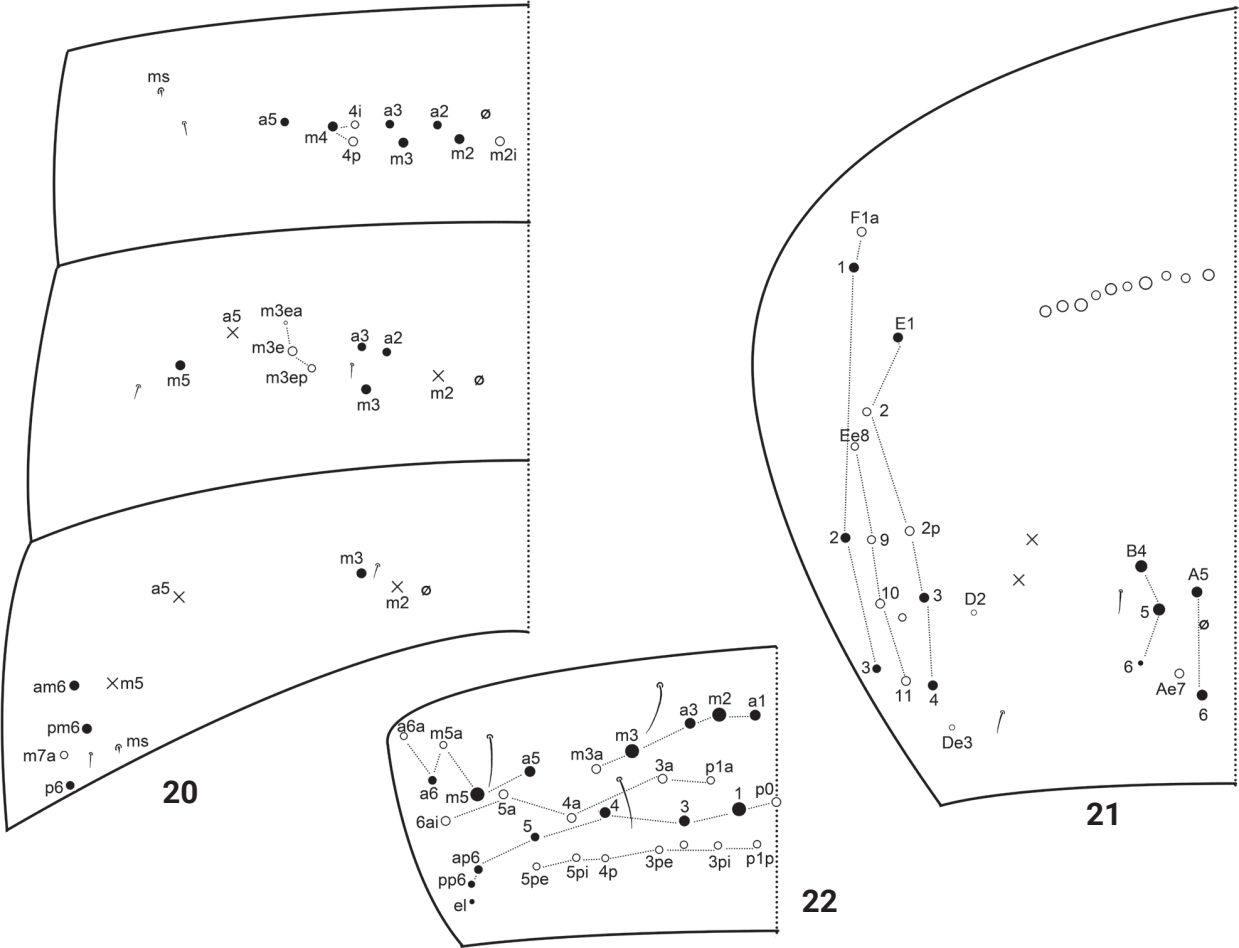
Abdominal chaetotaxy of *Homidiacingula***20**Abd. I–III **21**Abd. IV **22**Abd. V.

Th. II with three (m1, m2, m2i) medio-medial, three medio-lateral (m4, m4i, m4p), about 23 posterior mac and three lateral s-chaetae; ms internal to al; m7 and p6 as mic. Th. III with about 30 mac and two lateral s-chaetae; a7 and m7 as mic (Fig. 19). Abd. I with nine (m2–4, m2i, a2, a3, a5, m4i, m4p) central mac and two lateral S-chaetae. Abd. II with five (a2, a3, m3, m3e, m3ep) central, one (m5) lateral mac and two S-chaetae; chaeta m3ea as mic. Abd. III with one (m3) central, four (am6, pm6, p6, m7a) lateral macrochaetae and three S-chaetae (Fig. 20). Abd. IV with 8–10 anterior mac arranged in a transverse row, 4–5 (A5, A6, B4, B5, Ae7) centrally posterior mac, about 15 lateral mac and 52–62 S-chaetae; mac Ae7 often absent; B6 as meso or mic (Fig. 21). Abd. V with three S-chaetae (Fig. 22).

#### Distribution.

India, Bangladesh, China (Yunnan), Thailand, Malaya, Singapore, Indonesia (Java, Sumatra, Sulawesi).

#### Remarks.

*Homidiacingula* is characterized by dark transverse bands on Abd. II–III, 5+5 central mac on Abd. II, and 1+1 central mac on Abd. III. It has similar colour pattern to *H.subcingula* from Dalat, Vietnam (dark Abd. II and III). Mitra (1976) considered them as synonyms and doubtfully thought that Börner’s *H.cingula* represented juveniles of *H.subcingula*. However, the re-examination of the type specimen (2.4 mm) of *H.subcingula* in MNHN by the senior author (F. Zhang) shows that both taxa are valid species, although the type of *H.cingula* is in poor condition and many details are impossible to visualize. *Homidiasubcingula* differs from the *H.cingula* in the narrow strip on posterior Abd. IV, Abd. V pigmented, 11+11 mac on Abd. I, and 6+6 (m3ea present) central mac on Abd. II. In addition, the validation of *H.glassa*, which also possesses pigmented Abd. II–III, is questionable. Chaetotaxy of Abd. I–III of *H.glassa* could be closer to *H.subcingula*, but this differs from that of *H.cingula* (Table 1).

**Table 1.** Morphological comparison of three *Homidia* species.

Characters	*H.cingula*	*H.subcingula*	*H.glassa*
**Middle patch on Th. II posteriorly**	Present	Absent	Absent
**Lateral stripe on Th. II**	Present	Absent	Absent
**Two small patches on Th. III**	Absent	Absent	Present
**Posterior band on Abd. IV**	Broad	Narrow	Absent
**Transverse band on Abd. V**	Absent	Present	Absent
**Mac on Abd. I**	9	11	10?
**Central mac on Abd. II**	5	6	6
**Central mac on Abd. III**	1	2	2
**Maximum body length (mm)**	2.38	2.40	2.64

##### ﻿Molecular results

**Figure 23.**
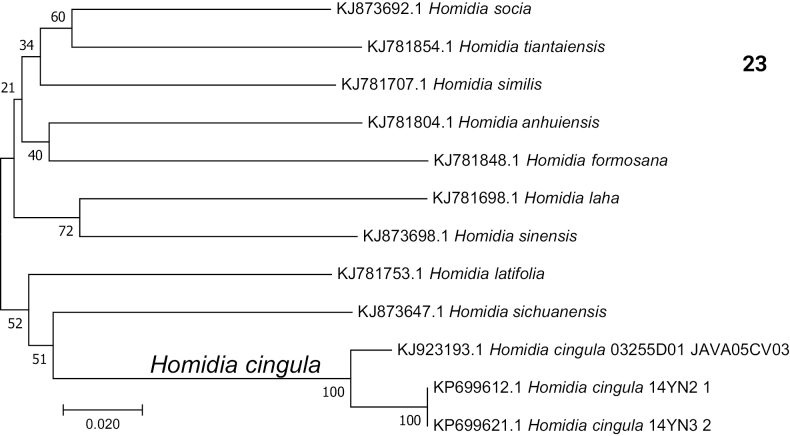
Neighbour-joining tree based on mtDNA COI sequences. Node numbers are bootstrap values.

Our results show that pairwise genetic distances range from 0.03 to 0.212 among 10 *Homidia* species (Table 2). The genetic distance between specimens of *H.cingula* from Yunnan (China) and Java is 0.03 (Table 2). The small genetic divergence (Hebert et al. 2003) indicates that these specimens belong to the same species (Fig. 23).

**Table 2.** K2P genetic distances among twelve *Homidia* sequences. YN, Yunnan; JAVA, Java.

GeneBank accession	Species	1	2	14YN2	14YN3	5	6	7	8	9	10	11
KJ781804.1	*Homidiaanhuiensis*											
KJ923193.1	*Homidiacingula*_03255D01_JAVA05CV03	0.205										
KP699612.1	*Homidiacingula*_14YN2_1	0.201	**0.03**									
KP699621.1	*Homidiacingula*_14YN3_2	0.201	**0.03**	0								
KJ781848.1	*Homidiaformosana*	0.174	0.191	0.204	0.204							
KJ781698.1	*Homidialaha*	0.212	0.193	0.198	0.198	0.206						
KJ781753.1	*Homidialatifolia*	0.17	0.161	0.173	0.173	0.197	0.187					
KJ873647.1	*Homidiasichuanensis*	0.173	0.157	0.173	0.173	0.204	0.186	0.163				
KJ781707.1	*Homidiasimilis*	0.158	0.188	0.209	0.209	0.19	0.192	0.161	0.179			
KJ873698.1	*Homidiasinensis*	0.173	0.169	0.185	0.185	0.175	0.158	0.196	0.183	0.157		
KJ873692.1	*Homidiasocia*	0.163	0.2	0.198	0.198	0.183	0.171	0.164	0.174	0.146	0.173	
KJ781854.1	*Homidiatiantaiensis*	0.17	0.192	0.199	0.199	0.184	0.203	0.172	0.167	0.157	0.176	0.138

## ﻿Discussion

Both morphological and molecular analyses confirm that the *Homidia* specimens from Indonesia and southwestern China, collected ca 3,000 km apart, are the same species. Species possessing distinct colour pattern (Abd. II–III and posterior half of Abd. IV pigmented) are widely distributed in Southeast and South Asia. Genetic divergence of the individuals from the most southern and the most northern regions is very low (ca 3%). Their colouration and wide distribution perfectly match the original descriptions and subsequent records of *H.cingula*. Therefore, we consider the species examined in this study to be *H.cingula*, although the type material described by Börner has been destroyed (Weidner 1962). Re-examination of types of *H.subcingula* verifies its validity based on colour pattern and chaetotaxy of Abd. IV (Table 1). The maximum body lengths of *H.cingula* and *H.subcingula* were approximately equal, thus disproving the hypothesis that *H.cingula* represents juveniles of *H.subcingula*. We doubt the validity of *H.glassa*, whose characteristics is very similar to *H.subcingula* except for its rough description of colouration. Applying colouration to distinguish *Homidia* species remains a powerful tool in modern taxonomy. Following this rule, the photographs of “*H.cingula*” from Taiwan (photographer: H.-J. Cheng) seem different from our understanding of *H.cingula*: both anterior and lateral margins are pigmented (anterior part pale in *H.cingula*), and the posterior half patch of Abd. IV is divided into two parts (connected in *H.cingula*). A molecular comparison could easily resolve this problem. Combining with the first instar chaetotaxy (Zhang et al. 2019), our revisiting of *H.cingula* provides valuable information for the diagnoses of the genus *Homidia*. Further collections of *H.cingula* from type locality (Buitenzorg, Indonesia) could be conducted to assign the neotype material.

## Supplementary Material

XML Treatment for Entomobrya kali

## ﻿Additional information

### Author ORCIDs

Feng Zhang https://orcid.org/0000-0002-1371-266X

### Data availability

All of the data that support the findings of this study are available in the main text.
